# Glycopeptidolipid Defects Leading to Rough Morphotypes of Mycobacterium abscessus Do Not Confer Clinical Antibiotic Resistance

**DOI:** 10.1128/spectrum.05270-22

**Published:** 2023-02-01

**Authors:** Yizhak Hershko, Amos Adler, Daniel Barkan, Michal Meir

**Affiliations:** a Clinical Microbiology Laboratory, Tel Aviv Sourasky Medical Center, Tel Aviv, Israel; b Koret School of Veterinary Medicine, Robert H. Smith Faculty for Agriculture, The Hebrew University of Jerusalem, Rehovot, Israel; c Sackler Faculty of Medicine, Tel Aviv University, Tel Aviv, Israel; d Ruth Rappaport Children's Hospital, Rambam Medical Center, Haifa, Israel; Johns Hopkins University School of Medicine

**Keywords:** *Mycobacterium abscessus*, glycopeptidolipid, antibiotics, resistance, rough, antibiotic resistance

## Abstract

Mycobacterium abscessus is an emerging pathogen causing severe pulmonary infections. Within chronically infected patients, M. abscessus isolates undergo molecular changes leading to increased virulence and antibiotic resistance. Specifically, mutations in glycopeptidolipid (GPL) synthesis genes, leading to the rough phenotype, are associated with invasive, nonremitting infections and a severe clinical course. It has been unclear whether GPL defects confer antibiotic resistance independently of other molecular changes. We used transposon technology to isolate a rough (GPL-defective; Tn *MABS_4099c*^ZeoR^) mutant and compare it to a fully isogenic parent strain (ATCC 19977) bearing wild-type zeocin resistance (WT^ZeoR^). Antibiotic susceptibility profiles of Tn_4099c^ZeoR^ and WT^ZeoR^ were tested and compared using the Sensititre RAPMYCOI antimicrobial susceptibility test plate. MICs were evaluated within clinically relevant values according to the Clinical and Laboratory Standards Institute (CLSI) standards. We found that M. abscessus with rough colony morphotype (Tn_4009c) had comparable antibiotic susceptibility to its smooth isogenic WT counterpart. Small differences (a 1:2 dilution) in MICs were found for imipenem, cefoxitin, and tigecycline, yet those small differences did not change the clinical susceptibility report for these antibiotics, as they fell within the same CLSI cutoffs for resistance. While small alternations in susceptibility to imipenem, cefoxitin, and tigecycline were noted, we conclude that the GPL mutations in M. abscessus did not confer clinically significant antibiotic resistance. Increased antibiotic resistance in the clinical setting may occur in an unrelated and parallel manner to GPL mutations.

**IMPORTANCE** Mycobacterium abscessus chronically infects patients with preexisting lung diseases, leading to progressive deterioration in pulmonary function. The common perception among clinicians is that the rough phenotype is associated with progressive disease and severe clinical course, manifested as a widespread inflammatory response and resistance to antibacterials. However, as clinical isolates accumulate hundreds of mutations over the prolonged course of infection, it is unclear whether the rough phenotype *per se* is responsible for the antibiotic resistance seen in late-stage infections, or whether the resistance is related to other genetic changes in the bacteria. Previous studies mostly compared rough and smooth clinical isolates. Here, for the first time, we compared WT smooth bacteria to a specific rough, GPL-associated, otherwise-isogenic mutant. We determined that the rough morphotype had essentially identical antibiotic susceptibilities as the parent strain. The mechanistic basis for the antibiotic resistance observed in rough clinical isolates is therefore most probably related to other genetic determinants.

## OBSERVATION

Mycobacterium abscessus is an emerging pathogen known to cause severe pulmonary infections among patients with cystic fibrosis (CF) and other chronic lung diseases (1, 2). In the clinical setting, M. abscessus appears as either smooth (S) or rough (R) colony variants, mostly associated with mutations in genes related to synthesis of the glycopeptidolipid (GPL) outer layer, such as inactivating mutations in the 20-kb GPL complex (*MAB_4098c* [*mps2*], *MAB_4099c* [*mps1*]) or GPL transport (*MAB_4115* [*mmpL4b*]) (3). Other mechanisms of the S-to-R transition have been described, but they are considerably rarer and often entail transcription factors affecting GPL synthesis as well as other cellular processes (4). Phenotypically, the S variant is known for its relative transmissibility, biofilm formation, and persistence, while the R variant is associated with an increased inflammatory response, extracellular chording, and hypervirulence, causing increased lethality in animal models and rapidly deteriorating lung functions in human patients (5–10). Microbiology studies examining antibiotic susceptibility of M. abscessus isolates of R and S morphotypes have noted mixed results (11, 12). While some studies reported similar susceptibilities, others noted R isolates to be more resistant, especially to beta-lactam antibiotics, such as imipenem and cefoxitin (13), leading to the prevailing paradigm that R isolates are necessarily antibiotic resistant. Within the human host, M. abscessus strains accrue multiple mutations leading to increased pathogenicity and increased antibiotic resistance (14). As these transformations occur in parallel to the transition from S to R variants, it has been unclear whether GPL synthesis defects *per se* reduce antibiotic susceptibility, independently of other molecular changes. To elucidate this point, we used our verified GPL-defective transposon mutant and an isogenic parent strain control to examine whether isolated GPL synthesis defects affect susceptibility to the antibiotics in clinical use in clinically relevant concentrations.

### Results.

Antibiotic susceptibility profiles of Tn_4099c and the wild-type zeomycin-resistant counterpart, WT^ZeoR^, were tested and compared using the Sensititre RAPMYCOI antibacterial susceptibility test (AST) plate. MICs were evaluated within clinically relevant ranges according to the Clinical and Laboratory Standards Institute (CLSI) standards (15) (Table 1). Both isolates were found clinically resistant to trimethoprim-sulfamethoxazole, doxycycline, and ciprofloxacin, with MICs above the CLSI breakpoints for resistance (exact MICs not determined). Both isolates were susceptible to amikacin (identical MICs of 16 μg/mL) yet resistant to moxifloxacin (identical MICs of 8 μg/mL) and to tobramycin (identical MICs of 16 μg/mL). Both had intermediate susceptibility to linezolid (identical MICs of 16 μg/mL) and induced clarithromycin resistance (identical MICs of 0.5, 8, and 16 μg/mL on days 3, 5, and 14 of incubation). Minor susceptibility differences in MICs were noted for imipenem, cefoxitin, and tigecycline. All three antibiotics showed an increase of a 2-fold dilution in the rough compared to its smooth counterpart (Table 1). These differences in MICs did not change the breakpoint interpretation of any of these antimicrobials. Finally, Tn_4099 and WT^ZeoR^ both had MICs above the limit of detection for minocycline, amoxicillin-clavulonate, ceftriaxone, and cefepime (exact MICs were not determined), all of which are not commonly used to treat mycobacterial infection yet are included in the Sensititre commercial panel.

**TABLE 1:** Antibiotic susceptibility of Smooth and Rough (GPL-defective) isogenic strains

Antibiotic	Published S, I, and R breakpoints (μg/mL)^*a*^	Determined MIC (μg/mL)	
		WT^ZeoR^	Tn_4099
Imipenem	≤4, 8–16, ≥32	8	16
Clarithromycin	≤2, 4, ≥8	0.5 (day 3), 8 (day 5), >16 (day 14)	0.5 (day 3), 8 (day 5), >16 (day 14)
Linezolid	≤8, 16, ≥32	16	16
Doxycycline	≤1, 2–4, ≥8	>16	>16
Tigecycline	≤0.5, 1–4, ≥8	0.12	0.25
Moxifloxacin	≤1, 2, ≥4	8	8
Ciprofloxacin	≤1, 2, ≥4	>4	>4
Tobramycin	≤2, 4, ≥8	16	16
Amikacin	≤16, 32, ≥64	16	16
TMP/SMX	≤2/38, NR, ≥4/76	>8/152	>8/152
Cefoxitin	≤16, 32–64, ≥128	32	64

a The susceptible (S), intermediate (I), and resistant (R) breakpoints for the indicated antibiotics. With the exception of tigecycline, breakpoint information was obtained from CLSI (15); the tigecycline breakpoints were previously published (26). TMP/SMX, trimethoprim-sulfamethaxazole; NR, not reported.

### Discussion.

M. abscessus isolates of rough colony morphotypes are often found in the clinical setting, especially in cases of chronic long-standing infection, and have been associated with severe clinical disease and worse clinical outcomes (5, 10). Antibiotic susceptibility studies of clinical isolates have also noted that rough colony clinical isolates have higher rates of resistance to clarithromycin, tigecycline, imipenem, and cefoxitin (11, 13). Within the human host, M. abscessus undergoes molecular changes that lead to increased pathogenicity (14) and increased antibiotic resistance. It is therefore unclear whether the GPL changes associated with the rough phenotype confer antibiotic resistance or, rather, that the genetic changes leading to the rough phenotype occur alongside the molecular changes leading to antibiotic resistance within chronically infected patients.

Our study is the first to describe the antibiotic susceptibility profile of isogenic smooth and rough M. abscessus. We found that rough GPL-defective M. abscessus isolates (Tn_4099) and their isogenic smooth counterparts had similar susceptibility results for most clinically used antibiotics. A minor, 1-fold dilution difference of MICs was noted for the beta-lactams imipenem and cefoxitin and for the tetracycline tigecycline, as previously described (11, 13). These differences did not change the breakpoint interpretations for these antibiotics. While these MIC differences may be of questionable clinical significance, they do raise questions regarding the possible effect of GPL synthesis on antibiotic susceptibility.

Antibiotic resistance mechanisms in M. abscessus are diverse and are often specific to the antibiotic group or mechanism of function. Resistance to beta-lactams is mostly mediated through beta-lactamases (16), while tigecycline resistance has been attributed to transcription modifiers (17). A common resistance mechanism may be related to cell wall permeability. GPL-deficient mycobacteria have been previously shown to have increased permeability to hydrophobic substances, suggesting that they would be likely to be more sensitive, rather than more resistant, to these substances. However, they did not appear to have altered MICs to hydrophobic or hydrophilic antibiotics (18). Mycobacterial efflux pumps have been associated with resistance to antibiotics such as bedaquiline, clofazemine, clarithromycin, amikacin, and tigecycline (19–22). Expression of efflux pump genes has been shown to be upregulated by antibiotic stress (22). It is unknown whether disabling MAB_4099c directly affects the regulation of efflux pump genes, yet it may be hypothesized that Tn_4099c may have increased cell wall permeability, allowing increased, albeit sublethal, exposure to the antibiotics, which may then lead to upregulation of efflux pump genes and thus paradoxical increased antibiotic resistance. Further studies are needed to evaluate the effect, if any, of MAB_4099c alterations on efflux pump gene expression.

It should be noted that the clinical outcome and treatment success of M. abscessus infections do not depend purely on the MIC. Indeed, M. abscessus is notorious for having discrepancies between the *in vitro* MIC and the clinical success of the treatment proposed on the basis of that MIC (1). Rough and smooth isolates may occupy different biological niches (intracellular, cavitary lesions, biofilm, thick mucus of patients with CF, etc.). The penetration of the antibiotic into each niche, the proliferation status of bacteria in them, and the degree of tolerance may differ (12). As the niche occupied by the pathogen may affect its physiology, the GPL (and other lipid) content may differ (23). Two isolates with similar *in vitro* MICs may therefore be differentially affected by the same antibiotic, making one clinically resistant and the other clinically susceptible. Minimal bactericidal concentrations (MBCs) may also differ in isolates with identical MICs. As MBCs are more complicated to test, most clinical laboratories do not report them, and clinical decisions are mostly based on MICs. Also, this study did not address the question of tolerance (i.e., the rate of killing) and the issue of persistence, and whether these characteristics were affected by GPL mutations. These are important questions that should be addressed in future studies.

A rough mutant may sometimes arise with no apparent mutation in the GPL pathway, probably related to a regulatory mutation affecting the expression of this pathway, but there are also other targets (3, 4). These isolates may possibly differ substantially in their MICs, and our conclusions here are restricted to specific defects in the GPL pathway.

In summary, M. abscessus of the rough colony morphotype has been found to have similar antibiotic susceptibility to its smooth isogenic counterpart. Small differences in MICs were found for imipenem, cefoxitin, and tigecycline, yet those compounds are of questionable clinical significance. Increased antibiotic resistance of rough clinical strains is therefore likely not related to the rough phenotype directly, but rather to additional molecular changes that occur within the chronically infected host.

### Materials and methods.

A *MAb_4099c* transposon mutant (Tn_4099) was isolated from our previously generated zeocin resistance-selected, M. abscessus ATCC 19977-based Tn mutant library (24), and it has been genetically characterized (25). A zeocin-resistant smooth counterpart (WT^ZeoR^) was created by insertion of a single-copy *attB*-integrating plasmid with zeocin resistance into M. abscessus ATCC 19977. Antibiotic susceptibility patterns of clinically used antibiotics were tested using the Sensititre RAPMYCOI AST plate (Thermo Scientific). Briefly, an inoculum suspension was prepared in demineralized water by gently scraping pure bacterial cultures grown on 7H10/glycerol agar plates, using sterile cotton swabs. The suspension was homogenized using the swab and passed through a 27-gauge needle to minimize clumping. A nephelometer was used to adjust the optical density to a McFarland 0.5 turbidity standard. Subsequently, 50 μL of the suspension was transferred to Müller-Hinton broth and tested with the Sensititre RAPMYCOI AST plate per the manufacturer’s instructions.
